# L-Arginine Activates the Neuregulin-1/ErbB Receptor Signaling Pathway and Increases Utrophin mRNA Levels in C2C12 Cells

**DOI:** 10.1155/bri/2171745

**Published:** 2025-03-07

**Authors:** Gladys Tapia, Sebastián Fuenzalida, Constanza Rivera, Pía Apablaza, Mónica Silva, Enrique Jaimovich, Nevenka Juretić

**Affiliations:** ^1^Programa de Farmacología Molecular y Clínica, Instituto de Ciencias Biomédicas, Facultad de Medicina, Universidad de Chile, Santiago 8380000, Chile; ^2^Programa de Biología Celular y Molecular, Instituto de Ciencias Biomédicas, Facultad de Medicina, Universidad de Chile, Santiago 8380000, Chile; ^3^Centro de Estudios de Ejercicio, Metabolismo y Cáncer, Programa de Fisiología y Biofísica, Instituto de Ciencias Biomédicas, Facultad de Medicina, Universidad de Chile, Santiago 8380000, Chile

**Keywords:** ADAM17, C2C12, L-arginine, Neuregulin, skeletal muscle

## Abstract

L-arginine induces the expression of utrophin in skeletal muscle cells, so it has been proposed as a pharmacological treatment to attenuate the symptoms of Duchenne muscular dystrophy (DMD). On the other hand, it has been described that one of the pathways that participates in the expression of utrophin in muscle is the Neuregulin-1 (NRG-1)/ErbB receptors pathway. Several studies have postulated that disintegrin and metalloprotease-17 (ADAM17) causes the proteolytic processing of NRG of transmembrane, allowing the release of NRG to the medium, which when joining its ErbB receptor activates the signaling pathway that triggers utrophin transcription. The aim of this study was to evaluate the effect of L-arginine in the activation of NRG-1/ErbB pathway and utrophin mRNA levels in C2C12 cells, and the participation of ADAM17 in this process. Our results indicate that L-arginine induces phosphorylation of ErbB2 and increases utrophin mRNA levels in C2C12 myotubes, with a maximum increase of 2-fold at 4 h post-stimulation. This effect is not observed when the myotubes are stimulated in the presence of GM6001 (general metalloprotease inhibitor) or PD-158780 (specific inhibitor of ErbB receptor phosphorylation). Experiments performed by flow cytometry suggest that L-arginine stimulates ADAM17 activation in our study model. Furthermore, immunofluorescence analysis supports our findings that L-arginine stimulates ADAM17 increase in treated myotubes. However, our results using pharmacological inhibitors suggest that ADAM17 does not participate in utrophin expression in C2C12 cells treated with L-arginine. The results obtained help to clarify the mechanism of action of L-arginine in the expression of utrophin in muscle cells, which will contribute to the design of new therapeutic strategies in pathologies such as DMD.

## 1. Introduction

Duchenne muscular dystrophy (DMD) is a severe muscle-wasting disease linked to X chromosome and has an incidence of 1 in 5000 born boys [1, 2]. DMD is caused by mutations associated with loss of function in the cytoskeletal protein dystrophin, which is normally expressed at the inner surface of the sarcolemma of muscle fibers [3].

Over the years, several therapeutic approaches have been studied, some of them pursuing restoration of dystrophin function [4] and others, stabilization and optimization of muscle structure [3], among these approaches, increasing endogenous utrophin levels appears promising [5]. Utrophin and dystrophin have functional redundancy and a high degree of homology towards the C-terminal domain [6].

Direct evidence for the ability of utrophin to functionally substitute dystrophin comes from experiments demonstrating that transgene-driven utrophin overexpression can effectively rescue dystrophin-deficient muscle in *mdx* mice [7, 8] and utrophin expression can be exogenously induced by gene [7] or pharmacological therapies [9].

A molecular mechanism described to increase utrophin expression is associated with Neuregulin (NRG) [10, 11], a protein secreted during muscle contraction [11]. NRG belongs to a family of proteins structurally related to the mitogen-epidermal growth factor (EGF), composed of 4 members (NRG-1 to NRG-4) [12, 13]. The best studied products are those encoded by NRG-1 gene. Furthermore, different isoforms are generated by alternative splicing of NRG-1 gene. The majority of NRG-1 isoforms have a transmembrane domain and an extracellular EGF-like domain, which is localized in the N-terminal portion [13] and could be released by metalloproteases (MMPs) and members of a disintegrin and MMPs (ADAMs) family like disintegrin and metalloprotease-17 (ADAM17), which has been mainly linked to NRG-1 shedding [14–16]. This allows for the linking of the bioactive domain of NRG-1 to its receptor [16]. NRG-1 binds to ErbB3 receptor, which does not have tyrosine kinase activity, and ErbB4, which has both binding and tyrosine kinase activity [13, 17]. When NRG-1 binds to either of the two ErbB receptors, a preferential heterodimerization is triggered with the orphan receptor ErbB2 [17], which do not contain a binding domain, but shows tyrosine kinase activity [13].

On the other hand, it has been described that L-arginine increases utrophin levels in the muscle. Moreover, experiments have demonstrated that L-arginine targets utrophin to the sarcolemma in dystrophic mice [18–20], suggesting a possible compensation for dystrophin loss in DMD [18].

Given this background and since the NRG-1/ErbB pathway and L-arginine are implicated in increasing utrophin expression in experimental models of muscular dystrophy [10, 11, 18–21], the aim of this study was to evaluate the effect of L-arginine in the activation of NRG-1/ErbB pathway and utrophin expression in C2C12 cells and to study the participation of ADAM17 in this process. We hypothesize that L-arginine increases utrophin expression in muscle cells via NRG-1β/ErbB in an (ADAM17)-dependent manner.

## 2. Methods

### 2.1. Materials

DMEM-F12 was purchased from Sigma (St. Louis, MO). Bovine serum and fetal bovine serum were purchased from GIBCO-BRL (Carlsbad, CA). Antibiotics and antimycotics were purchased from Life Technologies (Burlington, ONT, Canada).

L-arginine was obtained from Sigma-Aldrich. GM6001 and PD-158780 were from Calbiochem (La Jolla, CA).

Antibodies directed against ErbB2 (Neu C-18; sc-284) and ErbB3 (C-17; sc-285) were obtained from Santa Cruz Biotechnology, Inc. Antibodies directed against phospho-ErbB2 (Tyr 877; #2241) and phospho-ErbB3 (Tyr 1289; #4791) were from Cell Signaling Technology. Antibody anti-α-actinin (sarcomeric; clone EA-53, #A7811) was from Sigma-Aldrich. Antibody directed against ADAM17 (ab39163) was obtained from Abcam (flow cytometry antibody), and antibody directed against ADAM17 (MA5-32572) was obtained from Invitrogen (immunofluorescence antibody). Secondary antibody goat anti-rabbit IgG-HRP (sc-2004) was from Santa Cruz Biotechnology, antibody goat anti-mouse IgG-HRP was obtained from Pierce, antibody Alexa Fluor 488-conjugated affinipure goat anti-rabbit IgG (H + L) was from Jackson ImmunoResearch Laboratories Inc. and Alexa Fluor 546 donkey anti-rabbit IgG (H + L) (A10040) from Molecular probes by Life Technologies. All other reagents were from Sigma or Life Technologies.

ADAM17 and ADAM10 inhibitor (GW280264X) and ADAM10 inhibitor (GI254023X), from AOBIOUS INC, were kindly donated by Dr. Ricardo Moreno (Pontificia Universidad Católica de Chile).

#### 2.1.1. Cell Cultures

A normal C2C12 mouse cell line was used. Cells were seeded in 60 mm culture plates with medium composed of DMEM-F12 (1:1), 10% bovine serum, 2.5% fetal bovine serum, antibiotics, and antifungals. To eliminate the remaining fibroblasts, 10 μM of Ara-C was added when myoblasts started to line up. To induce differentiation, cells were seeded in serum-free medium, obtaining well-formed myotubes with spontaneous contractile activity between the 6th and 7th days.

#### 2.1.2. L-Arginine Treatment

C2C12 myotubes were exposed to 5 mM of L-arginine (Sigma-Aldrich) for different times [22]. When pharmacological inhibitors will be used, cells were preincubated for 30 min in resting medium with inhibitors prior to L-arginine incubation.

#### 2.1.3. RNA Extraction and Semiquantitative RT-PCR

Total RNA from C2C12 myotubes were prepared by Trizol Reagent extraction and reverse transcribed by using SuperScript II Reverse Transcriptase (Invitrogen). cDNA was amplified by PCR using mouse-specific primers for utrophin. DNA concentration was normalized to glyceraldehyde-3-phosphate dehydrogenase (GAPDH) expression.

The primers used were:

  Utrophin primers (548 bp):
  5′-GGGGAAGATGTGAGAGATTT-3′ (sense)
  5′-GTGTGGTGAGGAGATACGAT-3′ (antisense)
  GAPDH primers (350 bp):
  5′-TCCGCCCCTTCCGCTGATG-3′ (sense)
  5′-CACGGAAGGCCATGCCAGTGA-3′ (antisense)

PCR amplification was maintained in the exponential phase for each product. PCR conditions were:

- Utrophin: one cycle of 94°C for 2 min, followed by 26 cycles of 94°C for 1 min, 60°C for 1 min, 72°C for 1 min, and a final cycle of 10 min at 72°C.
- GAPDH: one cycle of 94°C for 2 min, followed by 21 cycles of 94°C for 30 s, 58°C for 40 s, 72°C for 1 min, and a final cycle of 10 min at 72°C.

PCR products were resolved by electrophoresis on 2% agarose gel and stained with ethidium bromide. Bands were quantified by densitometric analysis with the Scion Image program from NIH.

#### 2.1.4. Real-Time PCR (qPCR)

cDNAs were amplified by PCR using specific primers for utrophin. Quantification of mRNAs will be based on CT values, which represent the PCR cycle at which an increase in reporter fluorescence above baseline signal can be detected. PCR amplification of the housekeeping gene GAPDH was performed as a control.

#### 2.1.5. Western Blot Analysis

Cultured myotubes were lysed in 40 μL of ice-cold lysis buffer containing 20 mM Tris-HCl pH 7.5, 1% Triton X-100, 1 mM EDTA pH 8.0, 1 mM EGTA pH 8.0, 20 mM NaF, 1 mM Na_4_P_2_O_7_ x 10 H_2_O, 10% glycerol, 140 mM NaCl, and a mixture of 1 M PMSF (Sigma), 100 mM Na_3_VO_4_ (Sigma) with 7% protease inhibitors (Calbiochem), as previously described [21].

The cell lysates were incubated on ice for 1 h and centrifuged to remove debris. Protein concentration of the supernatants was measured using bovine serum albumin (BSA) as standard. 100 μg of lysate proteins were suspended in Laemmli buffer, separated in 7% SDS-polyacrylamide gels, and transferred to polyvinylidene difluoride (PVDF) membranes (Millipore).

Membranes were blocked in Tris-buffered saline containing 0.1% Tween 20 (TBS-T) and 5% BSA at room temperature, PVDF membranes were incubated overnight at 4°C with specific primary antibody. Membranes were then incubated with peroxidase conjugated secondary antibody for 1 h at room temperature. Proteins were visualized using an enhanced chemiluminescence system (Ez-ECL Kit, Biological Industries). The films were scanned, and the Image J program was employed for densitometric analysis of the bands. To correct for loading, the membranes were stripped with a solution containing 0.2 M glycine and 0.05% Tween pH 2.0 or ponceau red solution at room temperature and reproofed with the corresponding control antibodies.

#### 2.1.6. Surface ADAM17 Detection by Flow Cytometry

Live cells were blocked with DMEM-F12 supplemented with 3% BSA for 1 h. Anti-ADAM17 antibody was diluted 1:100 in the same blocking buffer and incubated at 4°C overnight, as previously described [23]. The cells were then washed 3 times in PBS and were incubated with anti-rabbit secondary antibody FITC conjugated for 1 h. Next, the cells were washed three times with PBS and the final pellet dissolved in PBS. The samples were analyzed by a flow cytometer (FACScanto, Becton-Dickinson) and 10,000 gated events were acquired in each sample. One sample was used as autofluorescence, while another was incubated with only primary antibody (anti-ADAM17 antibody ab39163; Abcam) and the third sample with only secondary antibody (Alexa Fluor 488-conjugated affinipure goat Anti-rabbit IgG (H + L); Jackson ImmunoResearch Laboratories Inc). All data was analyzed using BD FACSDiva Software.

#### 2.1.7. Immunostaining and Confocal Imaging

C2C12 myotubes were grown on coverslips. The myotubes were fixed in 4% paraformaldehyde in PBS, blocked in PBS containing 1% BSA for 60 min, and incubated with primary antibody at 4°C overnight (1:100; ADAM17 recombinant rabbit monoclonal antibody MA5-32572; Invitrogen). The cells were washed three times with PBS/BSA and incubated with secondary antibody at room temperature for 90 min (1:500; Alexa Fluor 546 donkey anti-rabbit IgG (H + L) A10040; Molecular probes of Life Technologies). The coverslips were mounted in Dako fluorescent mounting medium to retard photobleaching. The samples were evaluated in a scanning confocal microscope (Carl Zeiss Axiovert 135 m-LSM Microsystem) and documented through computerized images.

#### 2.1.8. Statistical Analysis

Data are represented as mean ± SEM of the number of independent experiments indicated (*n*) or as examples of representative experiments performed on at least three separate occasions. Data were analyzed by ANOVA and comparisons between groups were performed using Student's *t* test for paired data and Dunnett's multiple comparison test. A value of *p* ≤ 0.05 will set as the limit of statistical significance.

## 3. Results

### 3.1. L-Arginine Induces ErbB2 Phosphorylation in C2C12 Myotubes

**Figure 1:**
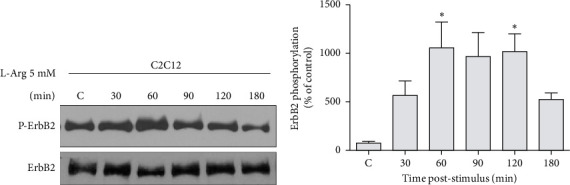
L-arginine induces ErbB2 phosphorylation in C2C12 myotubes. (a) C2C12 myotubes were incubated with 5 mM L-arginine (L-Arg) for different times. Proteins were extracted from these cultures, resolved on 7% SDS-polyacrylamide gels, transferred to PVDF membranes, and analyzed by Western blot using anti-phospho ErbB2 and anti-ErbB2 antibodies. Representative gels (*n* = 3). (b) Results normalized to ErbB2 expression and expressed as a percentage of nonstimulated control at 180 min (C = control; 100%). Bars represent mean ± SEM (*n* = 3). ⁣^∗^*p* ≤ 0.05 ANOVA followed by Dunnett's multiple comparison test.

To evaluate the effect of L-arginine on the activation of ErbB receptors, myotubes were stimulated with 5 mM L-arginine for different times (C, 30, 60, 90, 120, and 180 min), and ErbB2 phosphorylation was evaluated by Western blot. Results were normalized to ErbB2. We observed that L-arginine induces a transient increase on ErbB2 phosphorylation in C2C12 myotubes. Maximal phosphorylation (1054 ± 272) was achieved 60 min after L-arginine stimulation and is maintained up to 120 min (*n* = 3; Figure 1). We observed similar effects when ErbB3 receptor phosphorylation was studied (data not shown).

### 3.2. L-Arginine Stimulates Utrophin Expression in C2C12 Myotubes

**Figure 2:**
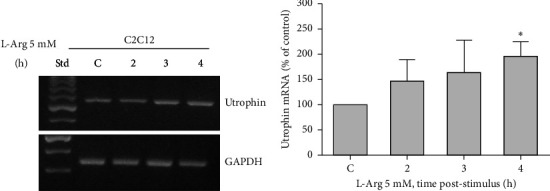
Effect of L-arginine on utrophin expression in C2C12 myotubes. (a) Total RNA was isolated from C2C12 myotubes stimulated with 5 mM L-arginine (L-Arg) for the times indicated. Utrophin mRNA level was analyzed by semiquantitative RT-PCR. Representative agarose gels of products from utrophin and GAPDH mRNA amplification. Std: standard molecular weight. (b) Results normalized to GAPDH expression and expressed as a percentage of nonstimulated control at 4 h (C = control; 100%). Bars represent mean ± SEM (*n* = 3). ⁣^∗^*p* ≤ 0.05 ANOVA followed by Dunnett's multiple comparison test.

To study the effect of L-arginine on the expression of utrophin in C2C12 cells, myotubes were stimulated with 5 mM L-arginine for different times (0, 2, 3, and 4 h) and the expression of utrophin was evaluated by semiquantitative PCR (RT-PCR; Figures 2(a) and 2(b)).

Our results show that L-arginine increases the level of utrophin mRNA at 4 h post-stimulus, with a maximum increase of 2 times.

### 3.3. Involvement of Metalloproteinase and ErbB Receptor Phosphorylation in the Upregulation of L-Arginine-Induced Utrophin in C2C12 Myotubes

**Figure 3:**
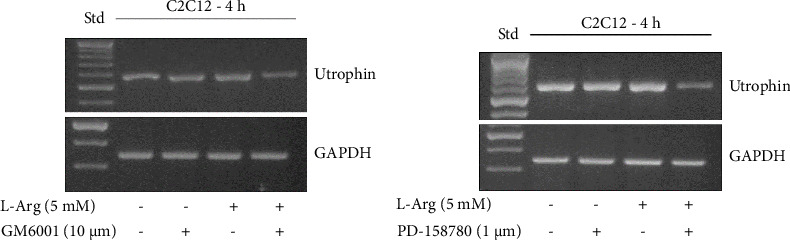
(a) Effect of GM6001 on L-arginine-induced utrophin expression in C2C12 cells. Myotubes were incubated with 5 mM L-arginine (L-Arg) in the presence of 10 μM GM6001 and vehicle (DMSO) for 4 h. Utrophin mRNA levels were analyzed by semiquantitative RT-PCR. Representative agarose gels of products from utrophin and GAPDH mRNA amplification (*n* = 3). (b) Effect of PD-158780 on L-arginine-induced utrophin expression in C2C12 cells. Myotubes were incubated with 5 mM L-Arg in the presence of 1 μM PD-158780 and vehicle (DMSO) for 4 h. Utrophin mRNA levels were analyzed by semiquantitative RT-PCR. Representative agarose gels of products from utrophin and GAPDH mRNA amplification (*n* = 3).

Although L-arginine stimulates utrophin expression in myotubes of C2C12 cells, this effect is not observed when myotubes are stimulated in the presence of a general MMP inhibitor (GM6001; Figure 3(a)) or a specific inhibitor of ErbB receptor phosphorylation (PD-158780; Figure 3(b)), suggesting the involvement of MMPs and ErbB receptor activation in this process.

### 3.4. L-Arginine Stimulates ADAM17 Activation in C2C12 Myotubes

To determine ADAM17 activation, its translocation to the cell surface was analyzed after 2 h of treatment with L-arginine, by flow cytometry.

**Figure 4:**
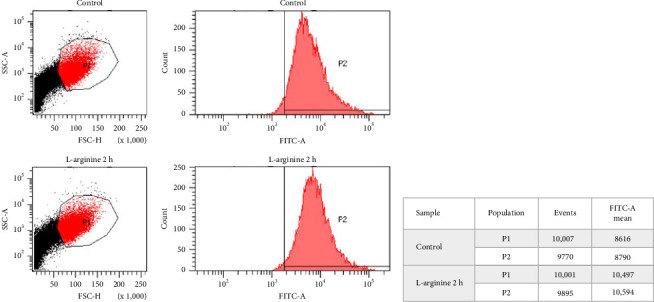
Effect of L-arginine on ADAM17 translocation to the cell surface in C2C12 cells. Results obtained by flow cytometry for control cells and those exposed for 2 h to L-arginine. (a) Delimitation of cellular P1, excluding remaining cellular debris by flow cytometry. (b) The cell quantity is observed on the vertical axis and the presence of FITC-A marker on the horizontal axis. (c) Fluorescence mean analysis performed by flow cytometry. The graphs shown correspond to a picture of a representative result (*n* = 4).

P1 represents the previously selected population where cellular residues were excluded. This was performed by analyzing the forward scatter height (FSC-H) and side scatter area (SSC-A) parameters of the cells comprising the control sample and the one exposed to L-arginine for 2 h (Figure 4(a)).

P2 represents the cell population that presents on its membrane surface the FITC-A marker bound to the protein of interest ADAM17 (Figure 4(b)). Our results show that in the control sample, approximately every 10,000 events a mean fluorescence of 8,790 was obtained. In the sample exposed to L-arginine for 2 h, a mean fluorescence of 10,594 was obtained. Therefore, an increase of 20.52% was observed with respect to the control sample (*n* = 4; *p* ≤ 0.05; Figure 4(c)), suggesting that L-arginine stimulates ADAM17 translocation to the cell surface at 2 h post-stimulation.

**Figure 5:**
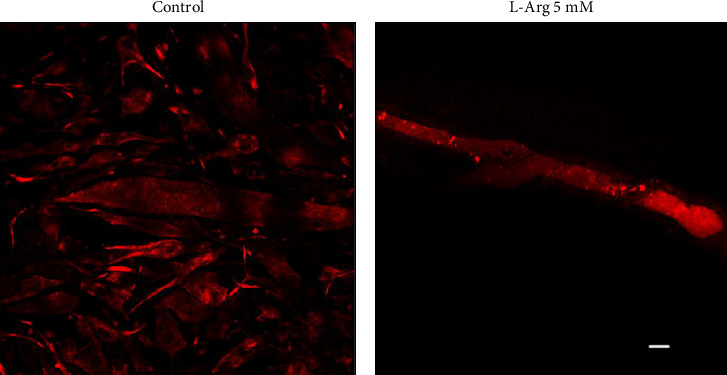
Immunostaining of ADAM17 in C2C12 myotubes. Analysis by immunofluorescence (a) control and (b) myotubes stimulated by 2 h with L-arginine 5 mM. Cells were fixed and incubated with anti-ADAM17 antibody (1:100) and then with secondary antibody Alexa Fluor 546 donkey anti-rabbit IgG (H + L) (1:500). Images were obtained by a confocal microscope (*n* = 3). Scale bar = 10 μm.

Furthermore, we performed immunofluorescence analysis of ADAM17 expression in C2C12 myotubes in basal conditions and at 2 h after L-arginine treatment (Figure 5). The samples were evaluated in a scanning confocal microscope (Carl Zeiss Axiovert 135 m-LSM Microsystem). We observed that in basal conditions, myoblasts appear highly labeled, while the stain in myotubes is faint. L-arginine treatment led to an induction in ADAM17 protein labeling. ADAM17 labeling is distributed throughout the cytoplasm and includes the borders (Figure 5), although it is not possible to assess whether L-arginine stimulates ADAM17 translocation to the cell surface at 2 h post-stimulation.

### 3.5. Participation of ADAM17 in Utrophin Expression in C2C12 Cells Treated With L-Arginine

To evaluate the participation of ADAM17 in the expression of utrophin stimulated by L-arginine, the myotubes were stimulated with L-arginine for 4 h in the presence and absence of an ADAM17 and ADAM10 inhibitor (GW280264X) and of ADAM10 inhibitor (GI254023X). Utrophin expression was evaluated by qPCR.

**Figure 6:**
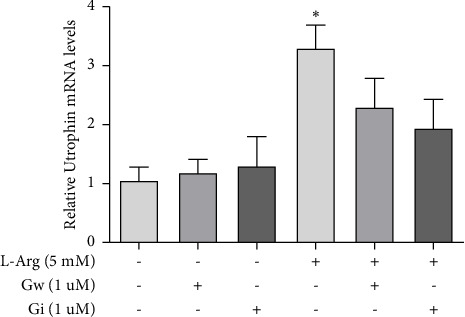
Effect of L-arginine on utrophin gene expression in the presence of ADAMs inhibitors in C2C12 cells. The C2C12 myotubes were stimulated for 4 h with 5 mM L-arginine in the presence or absence of ADAM17 and ADAM10 inhibitors (GW280264X; Gw) and only ADAM10 (GI254023X; Gi). Then, using qPCR, the results were evaluated by the delta method Ct (2^−ΔΔCt^) between the average Ct of the gene of interest (utrophin) and the housekeeping gene (GAPDH). The results were expressed as a percentage of the corresponding nonstimulated control (4 h: 100%). Bars represent mean ± SEM (*n* = 3). ⁣^∗^*p* ≤ 0.05 versus correspondent control, ANOVA followed by Dunnett's multiple comparison test.

Our results show that L-arginine increases relative utrophin mRNA levels at 4 h post-stimulus, with a maximum increase of 3.3-fold with respect to the control without stimulation (*n* = 3). Furthermore, these data suggest that ADAM17 does not participate in utrophin expression in C2C12 cells treated with L-arginine (*n* = 3) since inhibiting ADAM10 appears to be sufficient to decrease utrophin expression (Figure 6).

## 4. Discussion

We have clearly demonstrated that L-arginine supplementation increases utrophin mRNA expression in C2C12 cells and that this process is mediated by MMPs and ErbBs receptor phosphorylation. This will be valuable information if the same mechanism is shown to be present in human adult skeletal muscle, considering the important role that utrophin production could have on Duchenne and Becker muscular dystrophies patients.

Muscular dystrophies are part of a variety of alterations that are associated with several gene mutations leading to progressive muscle weakness and atrophy. DMD is an X-linked recessive disorder caused by a mutation in the dystrophin gene. Its worldwide incidence is estimated at 1/3500 births [20].

Dystrophin is a cytoskeletal protein normally expressed at the inner surface of the sarcolemma of muscle fibers [24], which is associated with a large complex of proteins known as the dystrophin-associated proteins (DAPs). Dystrophin with DAPs forms a link between the extracellular matrix (ECM) and the intracellular actin cytoskeleton, providing structural integrity to muscle fibers [6, 25]. Lack of dystrophin in muscle results in loss of DAPs and impairs the plasma membrane stability, causing mechanical stress and fragility [6, 25].

Utrophin is a protein structurally homologous to dystrophin. Several studies have shown that the replacement of dystrophin by utrophin could alleviate the symptomatology of DMD [26], making the latter a promising therapy. Moreover, studies in *mdx* mice have established that elevation of utrophin levels in muscle fibers can restore sarcolemma association of DAPs members and alleviate the dystrophic phenotype [5].

On the other hand, it has been described that the amino acid L-arginine induces an increase in utrophin expression in mouse muscle sarcolemma [18]. It has also been shown that NRG-1β activates a signaling pathway leading to increased utrophin transcription in muscle cells [21].

The aim of this study was to evaluate the effect of L-arginine on the activation of ErbBs receptors, utrophin expression and the involvement of ADAM17 in this process, as well as to investigate the possible functional relationship between L-arginine, NRG-1, ADAM17, and utrophin in C2C12 cells.

Our results indicate that L-arginine induces phosphorylation of ErbB2 and suggests the involvement of the ErbB3/ErbB2 heterodimer. This is in agreement with works describing that NRG stimulates tyrosine phosphorylation of ErbB2 and ErbB3, indicating that NRG signaling in skeletal muscle may be mediated by a complex of ErbB2 and ErbB3 [27]. However, future experiments will be necessary to study the effect of L-arginine on ErbB4 phosphorylation.

It has been described that NRG-1 pathway offers a potential mechanism to modulate utrophin expression in muscle. NRG-1 treatment of mouse and human cultured myotubes increases utrophin mRNA levels and induces utrophin transcriptional activity [10, 11]. Analysis of utrophin-A promoter region, isoform found in skeletal muscle fibers, revealed that it contains an N-box motif [28], which is targeted by the transcription factors known as GA binding protein *α* (GABPα) and β (GABPβ) [10, 11]. NRG-1 stimulates utrophin transcription through activation of pathways that phosphorylate GABPα and GABPβ, leading to their binding to the N-box motif [10, 11]. Moreover, we have recently demonstrated that NRG-1β is a regulator of utrophin expression, through GABPα transcription factor activation, in *mdx* myotubes [21].

On the other hand, Krag et al., have described that intraperitoneal injections of a small peptide region of the NRG-1 ectodomain to *mdx* mice increases utrophin expression and ameliorates the dystrophic phenotype [29]. Thus, NRG/ErbB signaling cascades could be potential therapeutic targets for DMD management.

Our results indicate that L-arginine increases utrophin mRNA levels in C2C12 myotubes; this effect is not observed when the myotubes are stimulated in the presence of a specific inhibitor of ErbB receptor phosphorylation (PD-158780). This is the first work that relates the effect of L-arginine with the activation of the NRG-1 signaling pathway and its relationship with the expression of utrophin in C2C12 myotubes. This positions L-arginine as a suitable candidate to treat DMD [30, 31] and to delay the onset of muscular dystrophy [31].

Some of the described benefits of L-arginine treatment are reduction of myonecrosis [20, 30], decreased creatine kinase serum levels and an increased isometric tension [20]. In addition, it leads to regeneration of affected muscles and a lower level of inflammation, mainly by downregulating the nuclear factor (NF)-κB/MMP cascade, decreasing the expression of several components, which explains the decrease in damage and necrosis in muscle cells [32].

Although the NRG-1/ErbB pathway would be playing a protective role, it is important to consider that NRG-1, upon binding to its receptor, can activate numerous signaling pathways, whose final effects may be deleterious. In this way, it has been described that treatment with ErbB2 targets has deleterious effects on cardiac function in patients with cancer, but nevertheless on the other hand, recent data suggest that NRG-1β could restore cardiac function after damage to the heart [33]. Other studies indicate that NRG-1 could be increased in cancer and neurological diseases, which makes it a pharmacological target [34]. Moreover, another study has implicated the ErbB4 receptor in actions associated with oncogenes and tumor suppressor genes, which could be due to the activation of different pathways or due to the expression levels of these receptors [35].

We propose that L-arginine contributes to the improvement of the dystrophic phenotype by increasing utrophin expression levels. However, studies show that the improvement can also be associated with an increase in the production of nitric oxide (NO) instead to utrophin upregulation. In fact, a recent report states that early administration of L-arginine in neonatal *mdx* mice ameliorated muscle performance without detectable utrophin increase and they attribute the observed effect to increase in NO production [31].

In this regard, metformin is a widely prescribed oral antidiabetic drug that has reached clinical trials for DMD in combination with the nitric oxide synthase (NOS) modulators L-arginine and L-citrulline (an L-arginine precursor). This drug increases skeletal muscle utrophin content via adenine monophosphate-activated protein kinase (AMPK) activation and parallel or reciprocal increments in PGC-1α and PPAR-δ expression [36]. Skeletal muscle nNOS activation is also AMPK dependent [37]. However, the partial response to metformin treatment in *mdx* muscles combined with the reduced quantity of NO in some studies supports the notion of combined therapy for DMD patients [36, 38]. In combination with L-arginine, metformin showed evident amelioration of muscular metabolism in the first proof-of-concept pilot study carried out in DMD patients [39]. Results from another study, a randomized, double-blind placebo-controlled clinical trial with 47 ambulant DMD patients, combining L-citrulline and metformin, showed a clinically relevant but not statistically significant reduction in motor function decline in a specific subgroup of patients with no apparent side effects [40]. Therefore, additional clinical trials are needed to validate this approach. It is important to consider then that L-arginine supplementation may be acting both through utrophin synthesis and by increasing NO production.

Considering that DMD is a chronic disorder, it could be useful to determine whether the prolonged stimulation of C2C12 with L-arginine is sufficient to sustain utrophin expression levels, and, on the other hand, if utrophin protein expression is stable over time. Therefore, further studies will be needed to determine protein expression levels of utrophin.

Moreover, given the possible translational effect of L-arginine treatment to induce the expression of utrophin to replace missing dystrophin, it would be important to know the expression and spatial location of the induced utrophin in C2C12 myotubes, perhaps along with members of the dystrophin in associated complex. We already know that utrophin upregulation by artificial transcription factors induces muscle rescue in *mdx* mice and locates in the sarcolemma [41].

ADAM17 and its close relative ADAM10 have been studied in the context of ectodomain shedding and have been demonstrated as key molecules in most of the shedding events [42]. ADAM17, also known as tumor necrosis factor-α converting enzyme (TACE), is a membrane-bound enzyme that sheds cell surface growth factor and cytokines, cytokine receptors extracellular domains, adhesion proteins, and ligands of ErbB (e.g., NRG) [42]. It has been described that this specific isoform is a molecular switch to control inflammation and tissue regeneration [43].

Experiments performed by flow cytometry suggest that L-arginine stimulates ADAM17 activation in our study model. Furthermore, immunofluorescence analysis supports our findings that L-arginine stimulates ADAM17 increase in treated myotubes. We observed that ADAM17 labeling clearly increased in the cytoplasm and may be also localized in the vicinity of sarcolemma. However, it will be interesting to validate these findings in cultured primary mouse muscle cells (myotubes) or adult muscle fibers.

Our results using pharmacological inhibitors suggest that ADAM17 does not participate in utrophin expression in C2C12 cells treated with L-arginine. Since in our study we used GW280264X (ADAM17 and ADAM10 inhibitor) and GI254023X (ADAM10 inhibitor), it will be important to perform experiments using an ADAM17-specific inhibitor. Furthermore, future studies should examine whether L-arginine treatment induces ADAM17-mediated proteolysis of NRG-1β.

On the other hand, our results show that treatment with a general metalloprotease inhibitor (GM6001) decreases utrophin gene expression in C2C12 cells stimulated with L-arginine.

Several studies suggest that gene expression and activation of various MMPs are dysregulated in patients with DMD [44]. Moreover, it has been postulated that elevated levels of MMPs may interfere with the process of muscle regeneration in dystrophic muscle [45, 46]. However, many studies have highlighted that not all MMPs are involved in the pathogenesis of muscular dystrophy and some of them may be activated as a part of a compensatory mechanism to counteract muscle damage [44]. Previous works have further revealed that MMPs could play critical roles in many physiological processes such as release of growth factors through shedding during tissue repair, among others [47]. Future studies should examine the type of MMP involved in the activation of the L-arginine-mediated NRG-1 pathway in muscle cells.

The results obtained help to clarify the mechanism of action of L-arginine in the expression of utrophin in muscle cells, which will contribute to the design of new therapeutic strategies in pathologies such as DMD.

## 5. Conclusions

L-arginine supplementation increases utrophin mRNA expression in C2C12 cells. This process is mediated by MMPs and ErbBs receptor phosphorylation, with a possible role of ADAM17.

The understanding of the molecular basis of regulation of NRG-1/ErbB pathway and L-arginine involvement will facilitate the design of new therapeutic strategies to potentiate muscle survival and regeneration in DMD patients.

## Data Availability

The data supporting the conclusions of this study are openly available. Please request information and access to the corresponding author.
