# Correction to “L‐Arginine Activates the Neuregulin‐1/ErbB Receptor Signaling Pathway and Increases Utrophin mRNA Levels in C2C12 Cells”

**DOI:** 10.1155/bri/9785358

**Published:** 2026-06-01

G. Tapia, S. Fuenzalida, C. Rivera, et al., “L‐Arginine Activates the Neuregulin‐1/ErbB Receptor Signaling Pathway and Increases Utrophin mRNA Levels in C2C12 Cells,” *Biochemistry Research International*, 2025, 2171745, https://doi.org/10.1155/bri/2171745.

In the article, there are errors in Figure 1, reported on PubPeer [1]. Specifically:

• In Figure 1, a section of the ERB2 bands appears extremely similar to the ERB2 bands in Figure 3b of [2], an article by the same authors
• In Figure 1, the p‐ERB2 blots appear highly similar to the p‐ERB2 blots from Figure 3b of [2], when compared with adjusted exposure and contrast

The authors contacted the Journal to correct the figure and further clarified that these errors were introduced during figure preparation. After assessment of the concerns and the author’s response, which included the original Western blots, the Editorial Board has determined that a correction to the Figure is appropriate. Please find the correct Figure 1 below:

**FIGURE 1:**
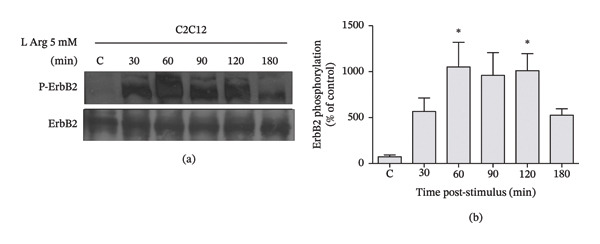
L‐arginine induces ErbB2 phosphorylation in C2C12 myotubes. (a) C2C12 myotubes were incubated with 5 mM L‐arginine (L‐Arg) for different times. Proteins were extracted from these cultures, resolved on 7% SDS‐polyacrylamide gels, transferred to PVDF membranes, and analyzed by Western blot using antiphospho ErbB2 and anti‐ErbB2 antibodies. Representative gels (*n* = 3). (b) Results normalized to ErbB2 expression and expressed as a percentage of nonstimulated control at 180 min (C = control; 100%). Bars represent mean ± SEM (*n* = 3). ^∗^ *p* ≤ 0.05 ANOVA followed by Dunnett’s multiple comparison test.

We apologize for these errors.
